# Evaluating the potential impact of targeted vaccination strategies against severe acute respiratory syndrome coronavirus (SARS-CoV) and Middle East respiratory syndrome coronavirus (MERS-CoV) outbreaks in the healthcare setting

**DOI:** 10.1186/s12976-019-0112-6

**Published:** 2019-10-07

**Authors:** Fatima Abdirizak, Rayleen Lewis, Gerardo Chowell

**Affiliations:** Department of Population Health Sciences, School of Public Health, Georgia State University, P.O. Box 3984, Atlanta, GA 30302-3984 USA

**Keywords:** MERS, SARS, Coronavirus, Nosocomial, Hospital transmission, Vaccine, Vaccination strategy, South Korea, Middle East, Stochastic simulation, And infection control and prevention

## Abstract

**Background:**

Severe Acute Respiratory Syndrome (SARS) and Middle East Respiratory Syndrome (MERS) are two coronaviruses with demonstrated potential to generate significant nosocomial outbreaks. In particular, MERS continues to pose a significant threat in the Middle East since 2012. Currently, no licensed vaccine or drug treatment is available to treat patients infected with either coronavirus. However, there are some MERS vaccines in the preclinical stage of development. We sought to evaluate the potential impact of targeted vaccination strategies for mitigating SARS and MERS outbreaks in healthcare settings using simple mathematical models and detailed historic transmission trees describing the progression of past nosocomial outbreaks of SARS and MERS.

**Results:**

Our findings suggest that vaccination strategies targeting patients and healthcare workers, which have been disproportionately affected during past outbreaks, and assuming two vaccination coverage levels at 50 and 75% have the potential to avert nearly 50% or more of MERS or SARS cases.

**Conclusion:**

Our modeling results informed by historic outbreak data for SARS and MERS suggest that vaccination strategies targeting patients could be an effective measure to mitigate and prevent outbreaks in the healthcare setting.

**Electronic supplementary material:**

The online version of this article (10.1186/s12976-019-0112-6) contains supplementary material, which is available to authorized users.

## Background

The 2003 outbreaks of Severe Acute Respiratory syndrome (SARS) were reported in 26 countries with a total of 8098 cases after 6 months [1, 2]. Although many countries reported cases of SARS, the disease was often limited to a few travel-related cases without any further subsequent spread [3]. However, five areas- Canada, China, Hong Kong, Singapore, and Vietnam- experienced substantial SARS outbreaks [3]. Sporadic importation of MERS, a related coronavirus, outside of the Middle East has primarily been due to returning travelers from the Middle East [4, 5]. Sustained MERS transmission outside of the Middle East was atypical until the South Korea outbreak, which became the largest MERS outbreak outside of the Middle East [5, 6]. The index patient in the South Korea outbreak developed MERS associated symptoms after returning from the Middle East [7]. After being discharged from the initial clinic he visited, he subsequently visited an emergency department in another hospital on the same day [7]. In the span of ten days, the index patient was seen in three hospitals [8]. By the end of the South Korea outbreak, there were 186 MERS cases involving 17 hospitals generated from a single transmission chain stemming from the index patient [8, 9].

Furthermore, Saudi Arabia has reported approximately 82% of MERS-CoV cases worldwide [5, 10]. The first MERS-CoV case was first identified in Saudi Arabia and has generated recurrent nosocomial outbreaks in the Middle East and one substantial outbreak in the Republic of Korea in 2015 [5]. Outbreaks in healthcare settings have been associated with overcrowding conditions, movement of undetected cases through the facility, and insufficient implementation of infection prevention and control measures [4, 5]. Additionally, the practice of seeking care at multiple health facilities, commonly referred to as “ hospital shopping”, is suspected to have contributed to the spread of MERS across varies hospitals in South Korea [7, 8]. The transmission dynamics of MERS-CoV outbreaks resemble those of the 2003-2014 SARS-CoV-a outbreaks in several areas of the world [11]. The modes of transmission and risk factors for MERS infection remain unclear. However, exposure to infectious camel or camel products appears to play an important role in triggering outbreaks [5, 12]. Thus, given the recurrent nature of MERS-CoV outbreaks in Saudi Arabia and the risk posed to other countries, it is important to understand the role of specific control interventions particularly in the healthcare setting [13–17].

Preventing and limiting the size of future outbreaks, especially of MERS-CoV, remains a priority for public health, and use of a vaccine in high-risk populations could be key to reduce associated mortality. Although SARS outbreaks have not been reported for 13 years, modeling SARS transmission and control in the healthcare setting could help devise control strategies for controlling MERS outbreaks, which are still occurring to date [5, 18]. SARS and MERS share some commonalities. Both diseases are notably seen to be amplified in healthcare settings and show to have some degree of transmission heterogeneity where superspreaders are a hallmark [11]. Currently, there are no reliable antiviral drugs or vaccines available for either coronaviruses, thus rapid diagnosis has been fundamental in managing outbreaks [19]. However, the lack of an appropriate animal model that mimics that natural history of the disease has slowed down the development of effective pharmaceutical interventions against MERS-CoV [20].

Once a MERS-CoV vaccine becomes available, it will be important to implement effective vaccination strategies, such as targeting those groups that generate the most MERS and SARS cases [21]. In this paper, we aim to model the potential impact of targeted vaccination strategies against hospital-based MERS and SARS transmission by using stochastic simulations and detailed transmission trees that describe the course of past MERS and SARS outbreaks in healthcare settings.

## Methods

**Fig. 1:**
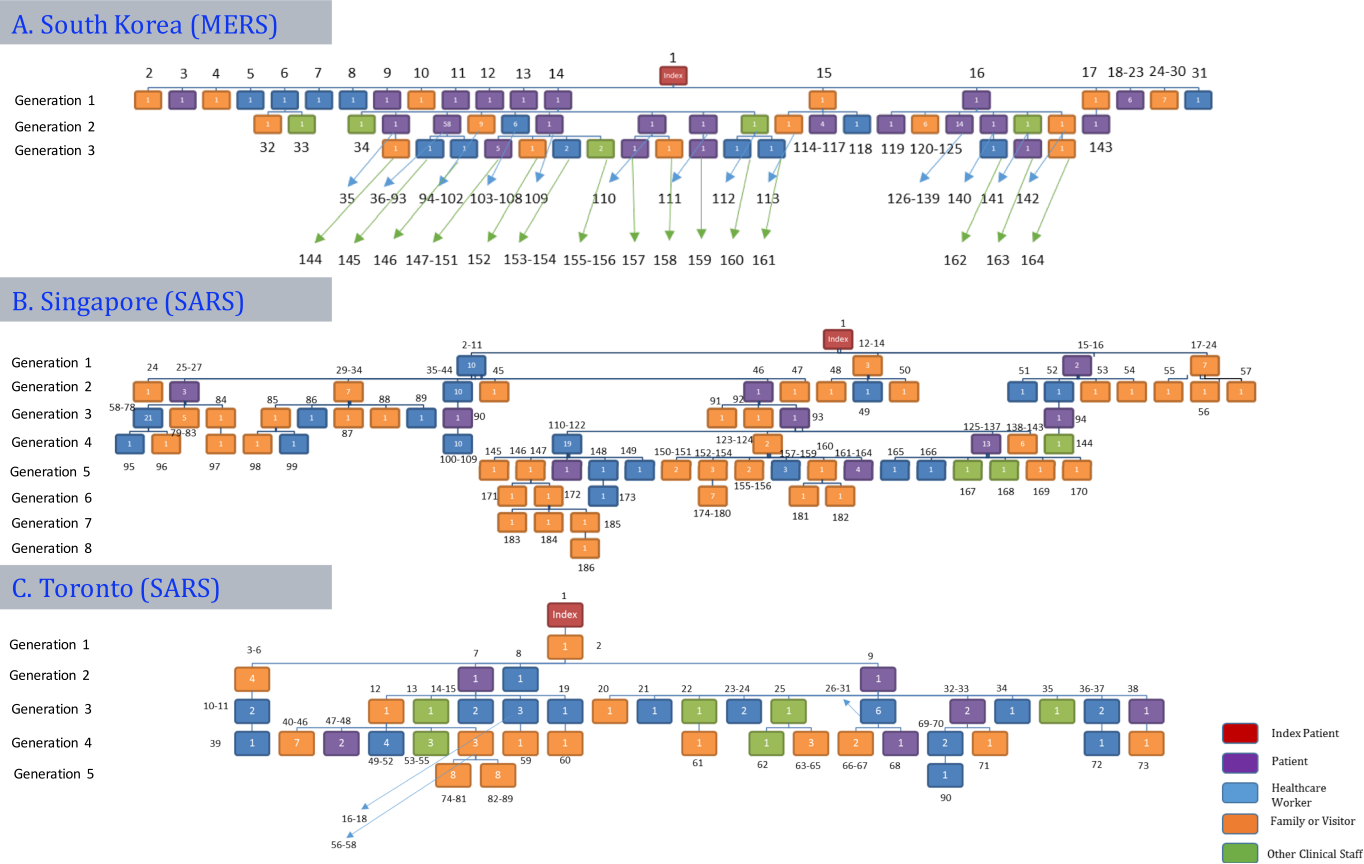
Transmission trees of Middle East Respiratory Syndrome and Severe Acute Respiratory Syndrome in healthcare settings. **a**. MERS outbreak in South Korea from May to July 2015 [10, 22–24]. **b**. SARS outbreak in Singapore from February to May 2003 [25]. **c**. SARS outbreak in Toronto from February to April 2003 [26]. The nodes in the transmission tree correspond to cases in the outbreak and the colors indicate the exposure category: patients, family/visitor, healthcare worker, and non-clinical staff

Our methodology to assess the impact of targeted vaccination strategies builds on prior modeling methods described in ref. [21]. In our study, we modeled the potential impact of targeted vaccination strategies on nosocomial outbreaks of MERS and SARS using transmission trees describing the temporal progression of past coronavirus outbreaks (Fig. 1). Our work expands the work in ref. [21] by providing simulation algorithms for generating multiple stochastic realizations to assess the effect of vaccination strategies using Monte Carlo simulation methods (Additional file 1).

### Data source

Transmission trees provide detailed information on the epidemiological links between cases, help identify super-spreaders, and highlight the duration of an outbreak in terms of disease generations. The transmission trees used in our analyses have been previously published in ref. [11]. The South Korea MERS outbreak took place in the summer of 2015 from May to July [22–24]. The transmission tree associated with this outbreak consists of 164 cases with 64% of those cases being patients (Fig. 1a) [22–24]. The SARS outbreaks in Singapore and Toronto occurred relatively around the same time in 2003 and unlike the MERS outbreak, most cases were among Healthcare workers (HCWs) and family/visitors [25, 26]. The transmission trees developed for these SARS outbreaks consist of 186 and 90 cases each for Singapore and Toronto (Fig. 1b-c) [25, 26]. Super-spreading events involve a single case, exposure to which results in a large number of secondary cases. Super-spreading events appeared to occur in the SARS and MERS outbreaks, with the number of cases resulting from each ranging from 8 to as many as 79 cases.

**Fig. 2:**
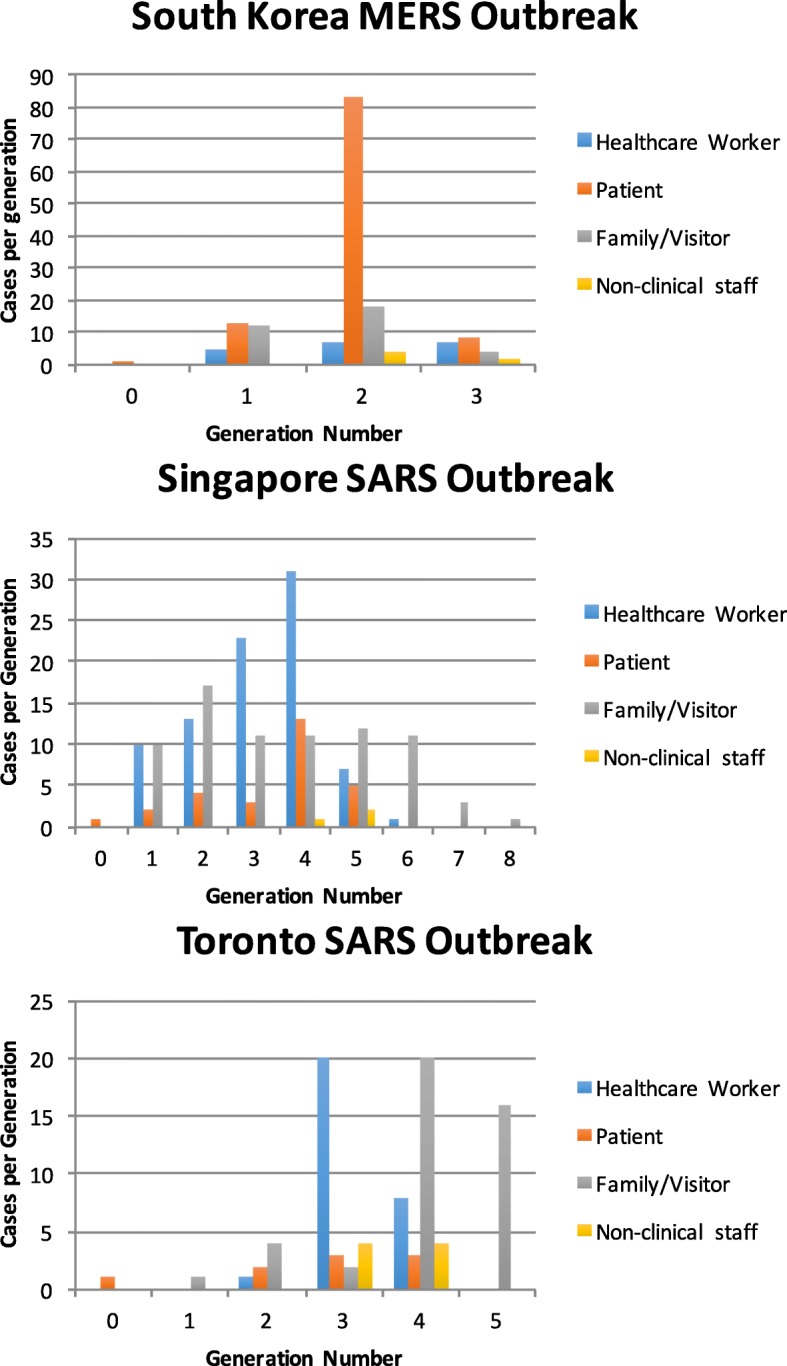
Total number of cases per generation for each exposure category (Healthcare worker, patient, family/visitor, and nonclinical staff) for MERS and SARS healthcare outbreaks

Here we assess vaccination strategies designed according to the distribution of cases among specific exposure categories in the healthcare setting: patients, healthcare workers, family or visitor, and other clinical staff (Table 1 & Fig. 2). In ref. [11], a comparative analysis on SARS and MERS outbreaks in healthcare settings revealed that MERS mostly affected patients whereas SARS greatly affected healthcare workers. Based on these findings, vaccination strategies were formulated by considering target population and vaccine coverage. For simplicity, here we assume that vaccine efficacy for each vaccination strategy is assumed to be 100%. Without loss of generality, vaccination coverage can also be interpreted as an “effective vaccination coverage” resulting from the product of vaccination coverage and vaccine efficacy.

**Table 1:** Total number of cases among various exposure categories for MERS and SARS outbreaks in healthcare settings

Outbreak	Type of Coronavirus	Time of Outbreak	Total Cases	Patients (%)	Healthcare Worker (%)	Family/Visitor (%)	Other Clinical Staff (%)	References:
South Korea	MERS	May ---July 2015	164	105 (64)	19 (12)	34 (21)	6 (4)	[10, 23, 24]
Singapore	SARS	February --- May 2003	186	28 (15)	79 (42)	76 (41)	3 (2)	[26]
Toronto	SARS	February --- April 2003	90	9 (10)	30 (16)	43 (23)	8 (4)	[27]

### Vaccine strategies

#### Vaccination strategy 1 (healthcare workers)

In this strategy, vaccination targets healthcare workers and assumes that vaccination covers 75% of healthcare workers all of whom are selected at random.

#### Vaccination strategy 2 (healthcare workers)

Vaccination targets healthcare workers, but the target vaccination coverage is lowered to 50%.

#### Vaccination strategy 3 (patients)

Patients have been seen to play a significant role in MERS transmission, which is most evident by inspecting the South Korea MERS transmission tree (Fig. 1). Thus, this strategy involves randomly vaccinating 75% of patients in the hospital.

#### Vaccination strategy 4 (patients)

In the case of MERS especially, individuals infected with MERS were older and likely to present with pre-existing conditions [11, 12, 14, 17, 22]. Since some patients may not be eligible to receive the vaccine, we also considered a lower vaccination coverage of 50%.

**Fig. 3:**
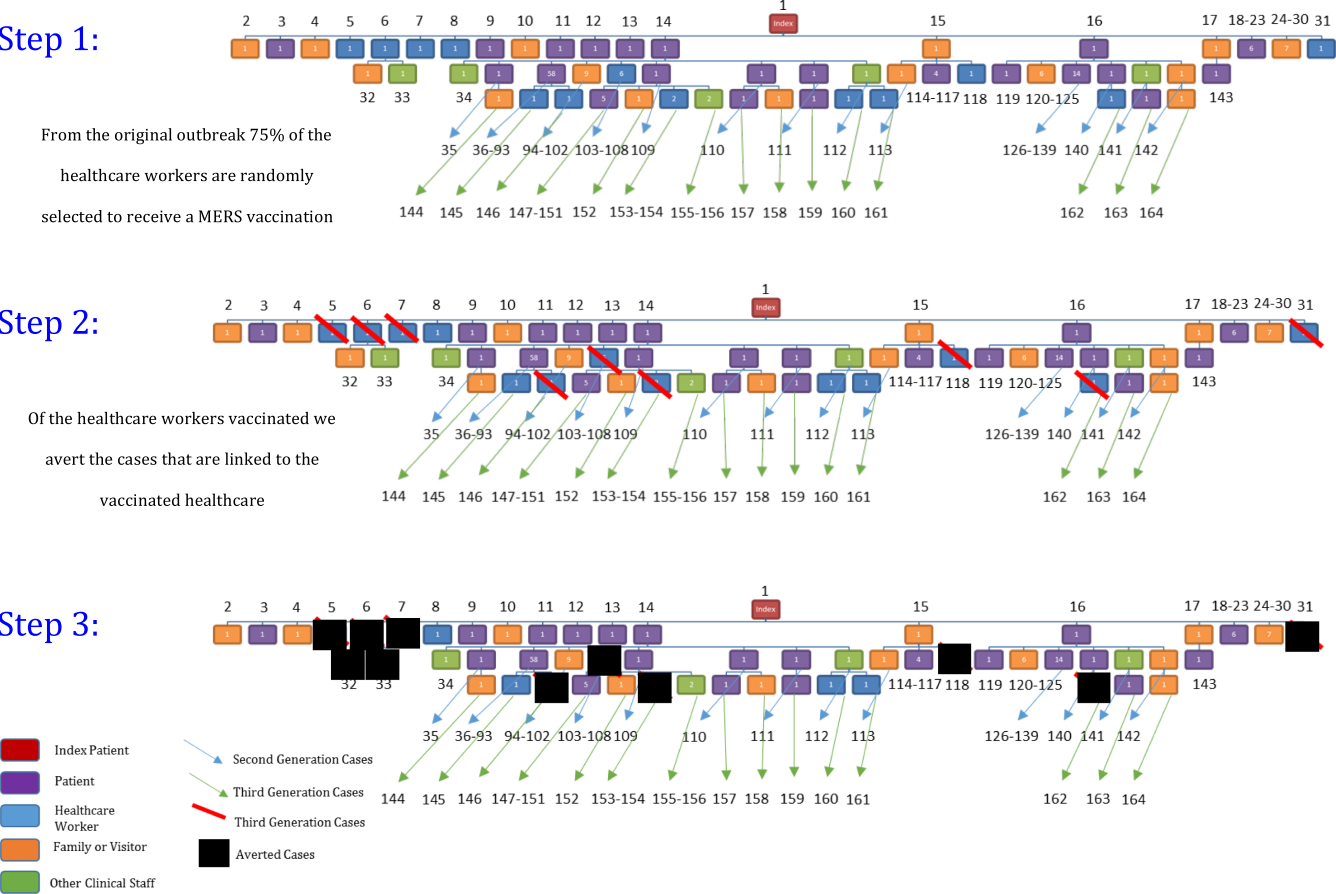
A vaccination strategy was modeled according to the following algorithm

Our algorithm employed to simulate the effects of vaccination strategies consists of the following four steps: (see Fig. 3).

Step 1: Individual Selection for Vaccination

Starting from a single transmission tree, the target individuals to be vaccinated are selected at random.

Step 2: Individual Vaccination

Once the individuals to be vaccinated are selected, those cases are automatically averted and removed from the outbreak (i.e., vaccine efficacy is 100%).

Step 3: Removal of Links

After averting the cases that have been vaccinated, all subsequent secondary individuals stemming from vaccinated cases are therefore considered averted.

Step 4: Repeat

In this study, we carried out 100 stochastic realizations of this vaccination process per transmission tree for each vaccination strategy. The algorithm was coded in R and is provided in the Additional file 1.

### Analysis

After the vaccination strategy was completed, we assumed that each person exposed to a case became infected. The proportion of cases averted for each simulation was calculated by dividing the number of cases averted by the total number of cases comprising the outbreak. The mean proportion of cases averted and corresponding 95% confidence interval using a z-distribution were calculated from 100 simulations. To create the graphs, 100 simulations of the vaccination strategy were run for a given vaccination coverage. For each simulation, the proportion of cases averted was calculated. The mean proportion of cases averted from the 100 simulations was recorded. This process was repeated for each vaccination strategy.

## Results

### Strategy 1: vaccinating 75% of HCWs

**Fig. 4:**
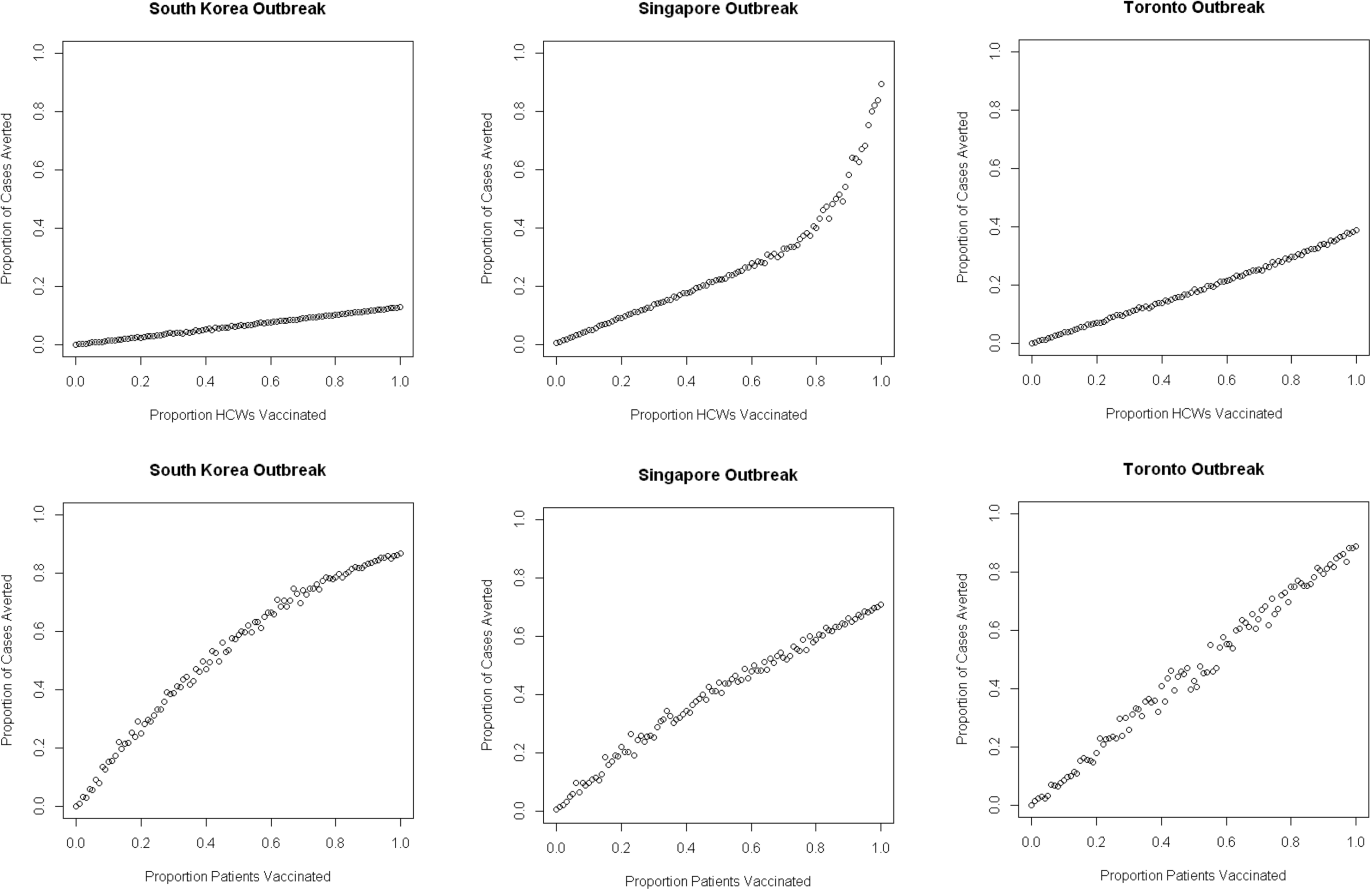
The proportion of cases averted per vaccine strategy in each Middle East Respiratory Syndrome (MERS) and Severe Acute Respiratory Syndrome (SARS) outbreak. The top panel illustrates the proportion of cases averted when HCW are vaccinated whereas the bottom panel demonstrates the proportion of cases averted when patients are vaccinated

Vaccinating 75% of HCWs appears to be a more effective strategy for SARS rather than for MERS. Only 10% (CI 4–16%) of cases were averted in the MERS outbreak. For Toronto, 27% (CI 18–36%) of the 90 cases were averted. Singapore would have benefitted the most from strategy 1, with a total of 36% (CI 26–45%) of cases averted (Table 2 & Fig. 4).

**Table 2:** Proportion of cases averted by each targeted vaccination strategies in each MERS and SAR healthcare setting outbreak

Outbreak	Vaccinated Population	Proportion Vaccinated	Proportion of Cases Averted (95% CI)
Singapore (SARS)	HCW	0.50	0.22 (0.14–0.30)
Singapore (SARS)	HCW	0.75	0.36 (0.26–0.45)
Singapore (SARS)	HCW	1.00	0.89 (0.83–0.95)
South Korea (MERS)	HCW	0.50	0.06 (0.01–0.11)
South Korea (MERS)	HCW	0.75	0.10 (0.04–0.16)
South Korea (MERS)	HCW	1.00	0.13 (0.06–0.19)
Toronto (SARS)	HCW	0.50	0.18 (0.10–0.25)
Toronto (SARS)	HCW	0.75	0.27 (0.18–0.36)
Toronto (SARS)	HCW	1.00	0.39 (0.29–0.48)
Singapore (SARS)	Patients	0.50	0.43 (0.34–0.53)
Singapore (SARS)	Patients	0.75	0.57 (0.47–0.67)
Singapore (SARS)	Patients	1.00	0.71 (0.62–0.80)
South Korea (MERS)	Patients	0.50	0.59 (0.49–0.69)
South Korea (MERS)	Patients	0.75	0.76 (0.67–0.84)
South Korea (MERS)	Patients	1.00	0.87 (0.80–0.93)
Toronto (SARS)	Patients	0.50	0.47 (0.37–0.57)
Toronto (SARS)	Patients	0.75	0.67 (0.57–0.76)
Toronto (SARS)	Patients	1.00	0.89 (0.83–0.95)

### Strategy 2: vaccinating 50% of HCWs

Reduction in coverage among HCWs inevitably averted fewer cases than strategy 1. In the South Korea transmission tree, reducing the vaccination coverage resulted in about a 50% decrease in the number of cases averted compared to strategy 1. Similar results were seen in the Singapore outbreak, only 22% (CI 14–30%) of cases were averted. In the Toronto outbreak, 18% (CI 10–25%) of cases were averted, which is only a 9% decline from the percent of averted cases in strategy 1 (Table 2 & Fig. 4).

### Strategy 3: vaccinating 75% of patients

Compared to vaccinating HCW, vaccinating 75% of patients averted more than 50% of cases in all of the outbreaks. For South Korea, 76% (CI 67–84%) of the cases were averted. Interestingly, vaccinating patients was also the most effective strategy in both SARS outbreaks. With this strategy, 57% (CI 47–67%) and 67% (CI 57–76%) of cases are averted respectively for South Korea, Singapore, and Toronto (Table 2 & Fig. 4).

### Strategy 4: vaccinating 50% of patients

Although vaccination coverage was reduced among patients, the percent of averted cases were either very close to 50% or much higher. Reducing vaccination coverage among patients resulted in a slight decline of 15, 6, and 18% for South Korea, Singapore, and Toronto in the total number of cases averted. Consequently, 61% of cases were averted for South Korea, 57 and 48% of cases were prevented for Singapore and Toronto (Table 2 & Fig. 4).

## Discussion

Our study provides the first analysis of coronavirus vaccine deployment strategies in the healthcare settings using simulations studies. Our modeling results indicate that for both viruses vaccinating at least 75% of patients yields a higher number of averted cases than any other vaccination strategy considered in our study. Although HCWs appear to be most affected by SARS, patients tend to infect the most people; therefore, vaccinating patients would achieve the greatest reduction in the number of HCWs infected. Additionally, for all the outbreaks the superspreaders were mostly patients and very few were family/visitors.

Furthermore, superspreaders are the hallmark of SARS and MERS transmission, which have been evident in the observed outbreaks (Fig. 1). For example, in South Korea, the index patient infected thirty individuals and in addition to two other patients collectively infected 75% of the cases involved in the outbreak [28]. Similarly, several super-spreading events occurred during the SARS epidemic. The index case in the Hong Kong outbreak was responsible for at least 125 cases and the same was observed at the Amoy Gardens housing complex and on the Air China flight [28]. Above all, early detection and compliance to infection control measures are fundamental in reducing the transmission of SARS but more importantly MERS, which still remains an issue [11, 28]. However, in the absence of such interventions, our study supports the deployment of vaccines targeting patients to lessen the risk of super-spreading events and ultimately avert the most cases.

Although patients play a prominent role in transmission in both SARS and MERS outbreaks, simply vaccinating all patients that enter a healthcare facility may be problematic and infeasible in some high-risk areas. Planning to vaccinate all patients is similar to implementing a national vaccination campaign. Additionally, patients have various lengths of hospital stay depending on the severity of their condition. A patient visiting an emergency room for a few hours may not have the same risk for MERS as a patient staying in the hospital for days or even months. It typically takes the body a few weeks to produce T-lymphocytes and B-lymphocytes after vaccination [29] so vaccinating patients during an outbreak may not be effective considering that immunity would not be built in time. We propose vaccinating patients with chronic diseases that require them to have multiple encounters with healthcare facilities such as those who are diabetic, have a respiratory illness, hypertension, or heart disease. For example, In Saudi Arabia with a population of roughly 30 million people, ~ 4.6 million annual visits are made to chronic disease clinics [30]. In the Al-Hasa outbreak, 52% of patients had end-stage renal disease, 74% had diabetes mellitus, 39% had cardiac disease, and 43% had lung disease [27]. In the Jeddah outbreak, 35% of patients had secondary exposure to MERS in the renal dialysis outpatient facility [14]. This evidence suggests a significant benefit in vaccinating patients with chronic diseases that put them at risk for MERS infection to ultimately reduce MERS transmission in healthcare settings.

There are limitations to this study. First, we only had access to a limited number of transmission trees for past outbreaks of MERS and SARS that include patients and healthcare workers. Having multiple transmission trees for MERS that capture the interaction between various exposure categories would provide additional evidence in determining the most effective vaccination strategy. Given the similarities between SARS and MERS transmission dynamics such as the superspreader events, we assessed the effects of vaccination against MERS transmission using SARS data. Third, since the transmission trees were extracted from multiple open-access sources and compiled by multiple individuals, completeness and effective contact tracing may have affected transmission patterns.

Our modeling results informed by real outbreak data support vaccinating patients primarily to prevent the most cases especially those with chronic diseases that put them at risk for MERS infection. Since there is still a significant need for more research on MERS vaccines, deployment of such a strategy currently is not plausible. Those infected with MERS tend to be older people with preexisting conditions such as diabetes, chronic lung disease, and cancer [31]. Thus, vaccinating patients with chronic illnesses may prove challenging and in the absent of a readily available vaccine, however, results from clinical trials would provide some insight into the matter. The potential impact of vaccines in the control of MERS will remain unknown until the vaccines under study move beyond the preclinical stage and into clinical trials. Considering that MERS is a continuing threat among the Gulf countries, the use of the Infection Prevention & Control Manual for GCC countries aids in the implementation of the first and second vaccination strategies across these countries in the Middle East, if HCW vaccination were to be undertaken. Again, before implementation, without an available vaccine for MERS to study, cost-effectiveness remains unknown. Without further research on the above concerns, the ultimate effect of vaccination is unclear; nonetheless deploying strategies to achieve an effective vaccination coverage among hospitalized risk groups appears to be critically needed for mitigating and preventing MERS outbreaks.

## Conclusion

With the use of stochastic simulations and detailed transmission trees of MERS and SARS nosocomial outbreaks, we explored the impact of targeted vaccination strategies and found that a vaccination strategy targeting 75% of the patients appeared to be the most effective. While sporadic MERS outbreaks have occurred due to diagnostic delays and lack of adherence to infection control measures which support super-spreading events, a vaccine may have a fundamental effect on reducing disease burden in these circumstances by preventing early transmission events and possibly reducing the risk of future MERS and SARS outbreaks in healthcare settings.

## Additional file

Additional file 1: The R code for the simulation algorithm for generating multiple stochastic realizations to assess the effect of vaccination strategies using Monte Carlo simulation methods is included as supplementary material. (R 7 kb)

## Data Availability

All data generated or analyzed during this study are included in this published article.

## Abbreviations

CI: Confidence interval
HCW: Healthcare Worker
MERS-CoV: Middle East Respiratory Syndrome- Coronavirus
SARS-CoV: Severe acute respiratory syndrome- coronavirus
